# Cyclic-di-GMP and *oprF* Are Involved in the Response of *Pseudomonas aeruginosa* to Substrate Material Stiffness during Attachment on Polydimethylsiloxane (PDMS)

**DOI:** 10.3389/fmicb.2018.00110

**Published:** 2018-02-01

**Authors:** Fangchao Song, Hao Wang, Karin Sauer, Dacheng Ren

**Affiliations:** ^1^Department of Biomedical and Chemical Engineering, Syracuse University, Syracuse, NY, United States; ^2^Syracuse Biomaterials Institute, Syracuse, NY, United States; ^3^Department of Biological Science, Binghamton University, Binghamton, NY, United States; ^4^Department of Civil and Environmental Engineering, Syracuse University, Syracuse, NY, United States; ^5^Department of Biology, Syracuse University, Syracuse, NY, United States

**Keywords:** *P. aeruginosa*, c-di-GMP, *oprF*, attachment, stiffness, mechanosensing

## Abstract

Recently, we reported that the stiffness of poly(dimethylsiloxane) (PDMS) affects the attachment of *Pseudomonas aeruginosa*, and the morphology and antibiotic susceptibility of attached cells. To further understand how *P. aeruginosa* responses to material stiffness during attachment, the wild-type *P. aeruginosa* PAO1 and several isogenic mutants were characterized for their attachment on soft and stiff PDMS. Compared to the wild-type strain, mutation of the *oprF* gene abolished the differences in attachment, growth, and size of attached cells between soft and stiff PDMS surfaces. These defects were rescued by genetic complementation of *oprF*. We also found that the wild-type *P. aeruginosa* PAO1 cells attached on soft (40:1) PDMS have higher level of intracellular cyclic dimeric guanosine monophosphate (c-di-GMP), a key regulator of biofilm formation, compared to those on stiff (5:1) PDMS surfaces. Consistently, the mutants of *fleQ* and *wspF*, which have similar high-level c-di-GMP as the *oprF* mutant, exhibited defects in response to PDMS stiffness during attachment. Collectively, the results from this study suggest that *P. aeruginosa* can sense the stiffness of substrate material during attachment and respond to such mechanical cues by adjusting c-di-GMP level and thus the following biofilm formation. Further understanding of the related genes and pathways will provide new insights into bacterial mechanosensing and help develop better antifouling materials.

## Introduction

Biofilms are communities of bacteria attached on surfaces and embedded in a self-produced matrix comprised of polysaccharides, DNA, and proteins (Arenas and Tommassen, 2016; Flemming et al., 2016). Biofilms of pathogenic bacteria cause serious chronic infections due to increased tolerance to antibiotics and host immune systems compared to their planktonic counterparts (Stewart, 2002; Hall-Stoodley et al., 2004). For example, the opportunistic pathogen *Pseudomonas aeruginosa* is a primary causative agent of chronic lung infections in cystic fibrosis patients, and is blamed for many other infections associated with chronic wounds and indwelling medical devices (Shirtliff and Leid, 2009; Hassett et al., 2010). A number of material properties influence biofilm formation (Song et al., 2015), such as surface chemistry (Cheng et al., 2007; Hou et al., 2009; Renner and Weibel, 2011), stiffness (Lichter et al., 2008; Saha et al., 2013; Guégan et al., 2014; Kolewe et al., 2015), hydrophobicity (Packham, 2003), topography (Hou et al., 2011; Singh et al., 2011; Crawford et al., 2012; Perni and Prokopovich, 2013), and charges (An and Friedman, 1998; Renner and Weibel, 2011). Among these properties, the effects of material stiffness are still poorly understood and previous studies are based on different materials with varying ranges of stiffness level, making it difficult to compare the results (Lichter et al., 2008; Saha et al., 2013; Guégan et al., 2014; Song and Ren, 2014; Kolewe et al., 2015). Thus, it is important to conduct systematic studies with consistent material properties. Recently, we reported that decrease in the stiffness of cross-linked poly(dimethylsiloxane) (PDMS) promotes the adhesion and growth of *Escherichia coli* and *P. aeruginosa*; and the attached bacterial cells on soft surfaces are longer and less tolerant to antibiotics (Song and Ren, 2014). These findings motivated us to further investigate what genes and pathways of *P. aeruginosa* are involved in its response to PDMS stiffness during attachment.

Several pioneering studies have explored how bacteria sense the general contact with a surface and transit from planktonic growth to biofilm formation (An and Friedman, 1998; Aprikian et al., 2011; Tuson and Weibel, 2013; Belas, 2014; Hughes and Berg, 2017). A number of genes related to flagella, pili, polysaccharides, and surface proteins have been shown to be involved in surface sensing by *P. aeruginosa*. It is known that motile bacteria can touch a surface with flagella to overcome the repellent force between cell body and the surface, and then use fimbriae to further secure the binding (Bos et al., 1999; Ploux et al., 2010). In this process, both flagella and pili are involved in surface sensing (Bhomkar et al., 2010; Aprikian et al., 2011; Petrova and Sauer, 2012). Increase in polysaccharide production leads to further biofilm formation. SadB and SadC were also found to regulate bacterial adhesion and motility (Merritt et al., 2007). The deletion mutants of *sadB* and *sadC* of *P. aeruginosa* PA14 exhibited marked defects in biofilm formation (Michael Dunne, 2002). Another mechanism of surface sensing through the Wsp pathway has also been identified in *P. aeruginosa* (Hickman et al., 2005). In this pathway, an inner membrane protein WspA has been found to be a receptor of signals associated with surface contact. Detection of surface attachment by WspA leads to phosphorylation of WspE. This results in an increase in the level of cyclic dimeric guanosine monophosphate (c-di-GMP), an important initiator of biofilm formation (O’Connor et al., 2012; Huangyutitham et al., 2013). C-di-GMP is a key factor regulating the transition from planktonic growth to biofilm formation; e.g., increase in the intracellular c-di-GMP level inhibits bacterial motility and promotes biofilm formation (Hickman and Harwood, 2008). The level of intracellular c-di-GMP is regulated by proteins containing GGDEF, EAL, or HD-DYP domain (Valentini and Filloux, 2016). For example, FleQ is a c-di-GMP responsive transcriptional regulator that binds to c-di-GMP and decreases the level of intracellular c-di-GMP (Hickman and Harwood, 2008). WspF is the inhibitor of WspR (DGC domain protein), the promoter of c-di-GMP synthesis (Hickman et al., 2005). Consistently, deletion of *fleQ* gene or *wspF* gene in *P. aeruginosa* led to increase in the level of intracellular c-di-GMP (Hickman and Harwood, 2008; Rybtke et al., 2012; Murakami et al., 2017).

Although the general sensing of surface contact and fluid shear by bacteria has been studied (Belas, 2014; O’Toole and Wong, 2016; Rodesney et al., 2017), how bacteria specifically sense and respond to material stiffness has only been scarcely explored and what genes are involved is largely unknown. Recently, we found that *E. coli* could actively respond to material stiffness during attachment, and *motB* is involved in such response (Song et al., 2017). Here, we show that *P. aeruginosa* mutants with inactivated *oprF*, *fleQ*, or Δ*wspF*Δ*psl*Δ*pel* lost the ability to differentiate material stiffness; and the intracellular level of c-di-GMP is important in regulating bacterial response to material stiffness.

## Materials and Methods

### Bacterial Strains and Growth Medium

The wild-type *P. aeruginosa* PAO1 and its isogenic mutants used in this study are listed in **Table 1**. All strains were routinely grown at 37°C in Lysogeny Broth (Bertani, 2004) (henceforth LB medium) containing 10 g/L tryptone, 5 g/L yeast extract, and 10 g/L NaCl in deionized (DI) water.

**Table 1:** List of *Pseudomonas aeruginosa* strains and plasmids used in this study.

*P. aeruginosa* strains or plasmids	Relevant genotype and/or characteristics	Source
***P. aeruginosa* strains**		
PAO1	Wild type	Jacobs et al., 2003
PAO1 *oprF*	*oprF* transposon mutant	Jacobs et al., 2003
PAO1 *motB*	*motB* transposon mutant	Jacobs et al., 2003
PAO1 *fliC*	*fliC* transposon mutant	Jacobs et al., 2003
PAO1 *pilA*	*pilA* transposon mutant	Jacobs et al., 2003
PAO1 *pelB*	*pelB* transposon mutant	Jacobs et al., 2003
PAO1 *pslD*	*pslD* transposon mutant	Jacobs et al., 2003
PAO1 *algC*	*algC* transposon mutant	Jacobs et al., 2003
PAO1 *oprE*	*oprE* transposon mutant	Jacobs et al., 2003
PAO1 *sadB*	*sadB* transposon mutant	Jacobs et al., 2003
PAO1 *sadC*	*sadC* transposon mutant	Jacobs et al., 2003
PAO1 *wspE*	*wspE* transposon mutant	Jacobs et al., 2003
PAO1 *wspR*	*wspR* transposon mutant	Jacobs et al., 2003
PAO1 *bifA*	*bifA* transposon mutant	Jacobs et al., 2003
PAO1 *rpoS*	*rpoS* transposon mutant	Jacobs et al., 2003
PAO1 *rpoN*	*rpoN* transposon mutant	Jacobs et al., 2003
PAO1 *rhlA*	*rhlA* transposon mutant	Jacobs et al., 2003
PAO1 σ^70^	σ^70^ transposon mutant	Jacobs et al., 2003
PAO1 *sigX*	*sigX*transposon mutant	Jacobs et al., 2003
PAO1 *fdxA*	*fdxA* transposon mutant	Jacobs et al., 2003
PAO1 *lecB*	*lecB* transposon mutant	Jacobs et al., 2003
PAO1 *mreC*	*mreC* transposon mutant	Jacobs et al., 2003
PAO1 *exoT*	*exoT* transposon mutant	Jacobs et al., 2003
PAO1 *fleQ*	*fleQ* transposon mutant	Jacobs et al., 2003
PAO1/pMH391		This study
PAO1*oprF*/pMH391		This study
PAO1 *oprF*/pMH391-*oprF*	*oprF* complement strain	This study
PAO1/pMH487		Rybtke et al., 2012
PAO1/pMH489		Rybtke et al., 2012
PAO1/pCdrA::*gfp*^s^	c-di-GMP reporter	Rybtke et al., 2012
PAO1/pCdrA::*gfp*(ASV)^s^	c-di-GMP reporter	Rybtke et al., 2012
PAO1 *oprF*/pCdrA::*gfp*(ASV)^s^	*oprF* mutant with c-di-GMP reporter	This study
PAO1/ Δ*wspF*Δ*psl*Δ*pel*Tn7CdrA::*gfp*(ASV)^c^	Δ*wspF*Δ*psl*Δ*pel* mutation strain with c-di-GMP reporter	Rybtke et al., 2012
PA14	Wild type	Liberati et al., 2006
PA14 *algU*	*algU* transposon mutant	Liberati et al., 2006
PA14 *dcgB*	*dcgB* transposon mutant	Liberati et al., 2006
**Plasmids**		
pMH391	Plasmid, Amp^r^, Gm^r^	Hentzer et al., 2002
pMH391-*oprF*	oprF complement plasmid, Gm^r^	This study
pMH487	Plasmid, empty vector for pCdrA::*gfp*^s^	Rybtke et al., 2012
pMH489	Plasmid, empty vector for pCdrA::*gfp*(ASV)^s^	Rybtke et al., 2012
pCdrA::*gfp*^s^	pUCP22Not-PcdrA-RBS-CDS-RNaseIII-gfp(Mut3)-T_0_-T_1_, Amp^r^, Gm^r^	Rybtke et al., 2012
pCdrA::*gfp*(ASV)^s^	pUCP22Not-PcdrA-RBS-CDS-RNaseIII-gfp(ASV)-T_0_-T_1_, Amp^r^, Gm^r^	Rybtke et al., 2012

### Preparation of PDMS Surfaces

Poly(dimethylsiloxane) surfaces were prepared using SYLGARD184 Silicone Elastomer Kit (Dow Corning Corporation, Midland, MI, United States). The stiffness was adjusted by varying the mass ratio of base to curing agent following a protocol described previously (Song and Ren, 2014). The base:curing agent ratios (wt/wt) of 5:1 and 40:1 were tested. For each given ratio, elastomer base and curing agent were thoroughly mixed and degased under vacuum for 30 min. Then, the mixture was poured into a Petri dish, cured at 60°C for 24 h, and incubated at room temperature for another 24 h to fully polymerize. The PDMS surface was then peeled off the Petri dish and cut into 1.0 cm by 0.6 cm pieces (1.5 mm thick), which were sterilized by soaking in 200 proof ethanol for 20 min and dried with sterile air. All of the sterilized PDMS substrates were stored at room temperature until use. The Young’s moduli of PDMS surfaces were measured using dynamic mechanical analysis (DMA) (Q800, TA instrument, DE, United States) as described in our previous study (Song and Ren, 2014).

### *P. aeruginosa* Adhesion on PDMS

*Pseudomonas aeruginosa* cells from overnight cultures were harvested by centrifugation at 8000 rpm for 3 min at 4°C, washed with phosphate buffered saline (PBS) (pH 7.3) three times, and diluted by PBS to the desired cell density. This cell suspension (30 mL) was transferred to a Petri dish containing sterilized PDMS surfaces. To exclude the effects of gravity, all biofilms were formed on face-down surfaces. After incubation at 37°C for 2 h without shaking, the PDMS surfaces were gently washed by dipping in PBS three times (changed to clean PBS for each step). The viability of cells was determined using the drop plate assay as described previously (Chen et al., 2003). Briefly, the attached cells were harvested by gentle sonication for 1 min and vortexing for 30 s. Then, the cell suspension was dropped on a LB agar plate after a series of 10× dilution (10 μL in each drop). The plate was included at 37°C overnight to count colony forming units (CFU). Each mutant was tested with duplicated samples in the screening. The positive results of *oprF*, *sigX*, and *mreC* mutants were each confirmed with at least three replicates.

Meanwhile, some PDMS surfaces were examined using an Axio Imager M1 fluorescence microscope (Carl Zeiss Inc., Berlin, Germany) to directly visualize the cells attached on PDMS surfaces. Acridine orange (500 μg/mL) was used to stain attached *P. aeruginosa* cells. At least five images were randomly taken from each sample, and the surface coverage by attached cells was calculated using COMSTAT (Heydorn et al., 2000). Thus, a total of 15 spots were randomly picked and analyzed from three replicate samples for each condition. The data of surface coverage and CFU were analyzed with *t*-test.

### Biofilm Growth

After attachment, the surfaces were washed three times with PBS to remove planktonic cells. The washed surfaces with attached cells were transferred to a new Petri dish containing 30 mL LB medium, and incubated at 37^o^C without shaking for 5 h. After incubation, the PDMS surfaces were gently washed and analyzed as described above. Surface coverage was determined using COMSTAT. The length of attached cells was measured directly from microscope images. At least 300 cells were analyzed for each condition. To understand if surface stiffness affects the growth of attached cells, the cells after 5 h of growth were stained with 500 μg/mL acridine orange in PBS for 2 min, and imaged with fluorescence microscopy. The same cells were also imaged using differential interference contrast (DIC) to corroborate the results.

### Tobramycin Susceptibility of Attached Cells

The washed surfaces after 5-h growth were transferred to a 12-well plate containing 2 mL PBS in each well supplemented with 20 μg/mL tobramycin, and incubated at 37°C without shaking for 3.5 h. The control surfaces were incubated under the same condition without antibiotic. After incubation, the number of viable cells was determined by counting CFU as described above. At least six repeats were tested in each condition.

### Genetic Complementation of the *oprF* Mutant

The *oprF* mutant of *P. aeruginosa* PAO1 was complemented with the plasmid pMH391 (Hentzer et al., 2002), which was obtained from Prof. Soren Molin at Technical University of Denmark. The *oprF* gene and its native promoters were amplified from the wild-type of *P. aeruginosa* PAO1 with primers 5′-CGCGGATCCTTGGGTAAATATTGTCTCTCT-3′ (forward primer with BamHI site) and 5′–CTAGTCTAGAAGGCTCAGCCGATTACTTGGC-3′ (reverse primer with XbaI site). The 1204 bp PCR product was inserted into the pMH391 vector between the BamHI and XbaI restriction sites to create pMH391-*oprF*. The new plasmid pMH391-*oprF* was transformed into the *oprF* mutant of *P. aeruginosa* PAO1 by electroporation and the right clones were selected with 50 μg/mL gentamicin.

### Measurement of Green Fluorescence in the c-di-GMP Reporter Strain Using a Fluorescence Spectrometer

This assay was used to monitor the c-di-GMP level in planktonic *P. aeruginosa* PAO1 and its mutants by following a protocol reported previously (Rybtke et al., 2012) with slight modifications. Briefly, *P. aeruginosa* PAO1 and mutants carrying pCdrA::*gfp*(ASV)^s^ were cultured overnight in LB medium supplemented with 60 μg/mL gentamicin. Subculture were made by inoculating LB medium with overnight culture to OD_600_ of 0.003. After OD_600_ reached 1.0, the cells were harvested by centrifugation at 6000 g for 3 min at 4°C and then washed with PBS (pH 7.3). The fluorescence of samples (150 μL culture each) in a black 96-well plate with transparent bottom was measured by a fluorescence plate reader (BioTek, United States). The cell densities were obtained by counting CFU and reading OD_600_. Each condition was tested with three replicates.

### Measurement of Green Fluorescence in the c-di-GMP Reporter Strain Using Fluorescence Microscopy and Flow Cytometry

This assay was used to monitor the c-di-GMP level in *P. aeruginosa* PAO1 cells attached on stiff or soft PDMS. Briefly, *P. aeruginosa* PAO1/pCdrA::*gfp*^s^ cultured overnight in LB medium supplemented with 60 μg/mL gentamicin was harvested by centrifugation at 6,000 g for 3 min at 4°C, washed with PBS (pH 7.3), and then used to inoculate 30 mL PBS with 60 μL washed overnight culture. This cell suspension (30 mL) was transferred to a Petri dish containing sterilized PDMS surfaces. After incubation at 37°C for 2 h without shaking, the PDMS surfaces were gently washed by dipping in PBS. Then the surfaces were imaged with fluorescence microscopy. Meanwhile, replicate samples were sonicated and vortexed to detach the cells as described above as controls, which were analyzed using a flow cytometer (BD Accuri C6, BD, United States). The results of cell density were corroborated by counting CFU as described above.

## Results

### Mutation of *oprF* Affected the Response of *P. aeruginosa* to PDMS Stiffness during Attachment

**FIGURE 1:**
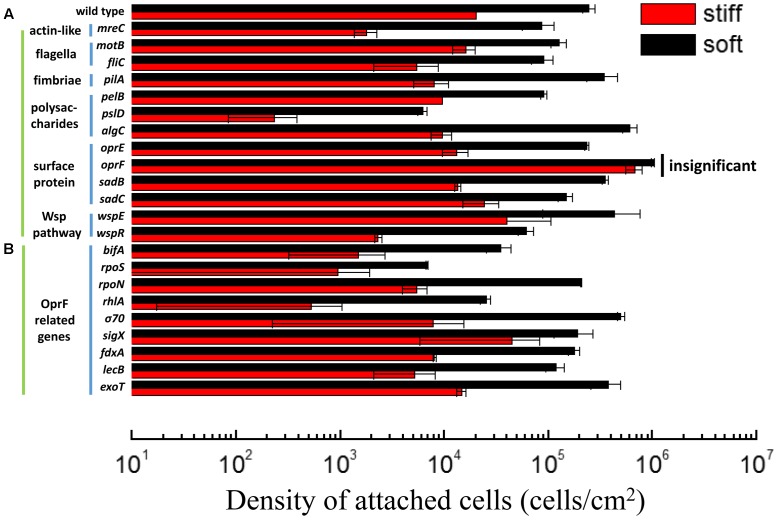
Adhesion of the wild-type *P. aeruginosa* PAO1 and its isogenic mutants on soft (40:1) and stiff (5:1) PDMS surfaces. All mutants showed significant difference in attachment between soft and stiff surfaces (*p* < 0.05, *t*-test), except the *oprF* mutant (labeled as insignificant). **(A)** The mutants of genes related to sensing surface contact. **(B)** The mutants of genes that have been reported to influence or be influenced by *oprF* expression. *rpoS*, *rpoN*, σ^70^, and *sigX* encode sigma factors.

We recently reported that the stiffness of PDMS has profound effects on the adhesion of *E. coli* and *P. aeruginosa*, as well as the growth, morphology, and antibiotic susceptibility of attached cells (Song and Ren, 2014). In agreement with our previous findings, there were (2.1 ± 0.7) × 10^5^ cells/cm^2^ wild-type PAO1 cells attached on 40:1 (soft) PDMS surfaces following 2 h post-initial adhesion, while only (1.8 ± 0.1) × 10^4^ cells/cm^2^ attached on 5:1 (stiff) surfaces (**Figure 1A**). Thus, when the stiffness increased from 0.1 to 2.6 MPa, the number of attached PAO1 cells decreased by 92 ± 3%. This is consistent with our previous report (Song and Ren, 2014). Several genes have been shown to be involved in general sensing of contact with a surface and the initiation of adhesion, such as those related to flagella, fimbriae, and the Wsp system (Hickman et al., 2005); however, how bacterial cells sense and respond to the stiffness of substrate material is still unknown. To understand how *P. aeruginosa* senses material stiffness, the above-mentioned attachment assay was used to screen several isogenic mutants of *P. aeruginosa* PAO1 (**Table 1**). All the tested mutants that are related to flagella, fimbriae, the Wsp system showed at least one log difference in the number of attached cells between soft and stiff PDMS surfaces, similar to the wild-type PAO1. This finding suggests that these genes are not critical for differentiating material stiffness by *P. aeruginosa*. This result also emphasizes that sensing material stiffness may require different system(s) than sensing the general contact with a surface.

Mammalian cells sense material stiffness using integrin and then transfer the signal to the nuclear envelop using myosin, actin, and nesprin (Wells, 2008; Wang et al., 2009). Actin is a key connector in the mechano-transduction and thus, sensing of material stiffness by eukaryotic cells (Ladoux and Nicolas, 2012). Several homologs of eukaryotic microfilaments (actin) have been identified in prokaryotic cells including *P. aeruginosa* such as *mreB* and *mreC* (Shaevitz and Gitai, 2010). To understand if *P. aeruginosa* uses a similar mechano-transduction pathway to respond to PDMS stiffness during attachment, 2 h adhesion of *mreC* mutant was exanimated using the same adhesion assay. The results show that the number of attached *mreC* mutant cells on soft surfaces was two logs more than those on stiff surfaces (*p* < 0.0001, *t*-test, *n* = 3), which is even more than the wild-type strain (less than two logs in difference). This indicates that the *P. aeruginosa mreC* mutant could still have different responses to the soft and stiff PDMS. Although this does not rule out the potential involvement of *mreC* in mechanosensing by *P. aeruginosa*, this gene is not essential to the response of *P. aeruginosa* to PDMS stiffness during attachment.

**FIGURE 2:**
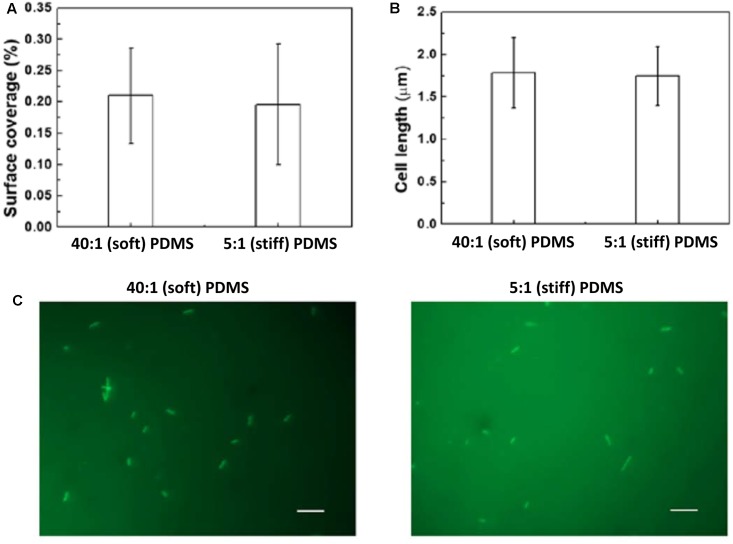
Effects of PDMS stiffness on growth and cell length of *oprF* mutant. No significant difference was observed between stiff and soft surfaces (*p* > 0.05 for both, *t*-test, *n* = 15 for surface coverage and *n* > 300 for cell length). **(A)** Surface coverage of attached cells calculated using COMSTAT. **(B)** Average length of attached cells on soft (40:1 PDMS) and stiff (5:1 PDMS) surfaces. **(C)** Representative images of 5 h biofilm cells strained with acridine orange (Bar = 10 μm).

In comparison, there were similar numbers of *oprF* mutant cells on the soft and stiff PDMS surfaces, e.g., (9.8 ± 0.03) × 10^5^ cells/cm^2^ on soft surface and (7.8 ± 0.7) × 10^5^ cells/cm^2^ on stiff surfaces (*p* > 0.05, *t*-test, *n* = 6). Since the attachment of wild-type *P. aeruginosa* PAO1 cells on stiff PDMS is 92 ± 3% less than that on soft PDMS, this finding suggests that *oprF* may be involved in response to PDMS stiffness. Because PDMS stiffness also affects the growth, morphology, and antibiotic susceptibility of attached *P. aeruginosa* PAO1 cells (Song and Ren, 2014), we speculated that mutation of the *oprF* gene may affect the difference in these phenotypes of *P. aeruginosa* between soft and stiff surfaces. To test this, the stiff and soft PDMS surfaces were incubated in PBS with 2 × 10^7^ *oprF* mutant cells/milliliter for 2 h to allow the cells to attach, and then transferred to LB medium to allow the cells to grow for 5 h. The surfaces were washed three times with PBS to remove planktonic cells before transfer. As shown in **Figure 2A**, after 5 h growth, the surface coverage of *oprF* mutant was similar between soft and stiff PDMS surfaces (*p* > 0.05, *t*-test, *n* = 15). Thus, mutation of *oprF* abolished the difference in growth of attached cells between soft and stiff surfaces.

Mutation of *oprF* also abolished the difference in cell length between soft and stiff PDMS surfaces exhibited by the wild-type strain. As we reported previously, the average length of attached wild-type *P. aeruginosa* PAO1 cells on soft PDMS surfaces was 1.6 times that of cells on stiff PDMS surfaces (Song and Ren, 2014). However, the average length of *oprF* mutant cells was about the same on soft and stiff PDMS surfaces, e.g., 1.76 ± 0.42 μm on soft PDMS and 1.70 ± 0.31 μm on stiff PDMS (*p* > 0.5, *t*-test; >300 cells counted for each condition; **Figures 2B,C**), which is between the cell lengths of the wild-type strain attached on soft (2.6 ± 0.07 μm) and stiff (1.4 ± 0.03 μm) PDMS surfaces (Song and Ren, 2014).

Previously we also found that the wild-type *P. aeruginosa* PAO1 cells on soft PDMS after 5 h growth (the cells were allowed to attach for 2 h in PBS first before switching to LB medium for biofilm growth for 5 h) are five times more susceptible to 20 μg/mL tobramycin in 3.5 h treatment than those on stiff PDMS (Song and Ren, 2014). Consistent with the changes in cell adhesion, growth, and cell size, it appears that *oprF* mutation strain also abolished the difference in antibiotic susceptibility of attached cells observed for the wild-type strain. This was supported by the finding of *oprF* mutant cells on soft and stiff PDMS exhibiting similar susceptibility to 20 μg/mL tobramycin (*p* > 0.05, *t*-test, *n* = 6; Supplementary Figure S1).

Collectively, these results indicate that *P. aeruginosa oprF* mutant lost the capability to respond to surface stiffness during attachment, and thus the differences in growth, cell morphology, and tobramycin susceptibility of attached cells between stiff and soft PDMS surfaces. OprF has been shown to be important for binding to animal cells including human lung epithelial cells (Azghani et al., 2002; Fito-Boncompte et al., 2011). The data obtained in this study indicate that *oprF* may also be involved in mechanosensing of material stiffness.

### Rescuing *oprF* Mutant with Genetic Complementation

**FIGURE 3:**
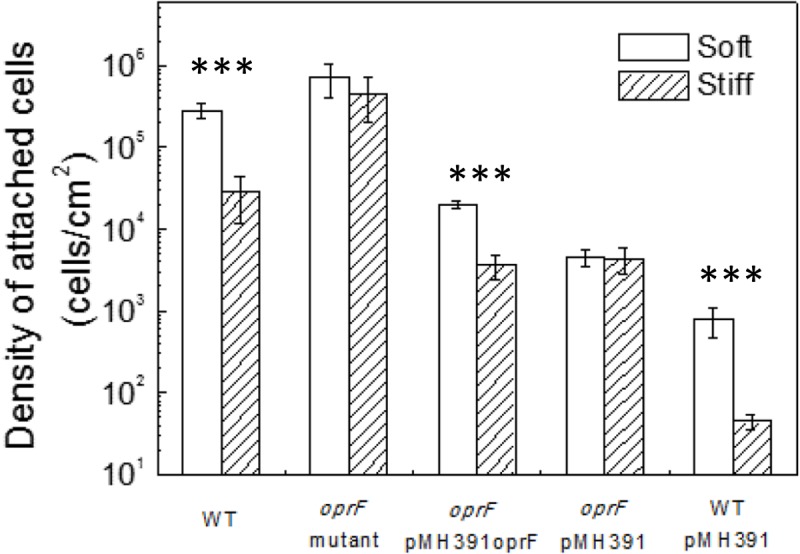
Effects of PDMS stiffness on the attachment of *P. aeruginosa* wild type, *oprF* mutant, the complemented strain, wild-type cells with empty vector, and mutant strain with empty vector. The number of attached cells on soft (40:1) and stiff (5:1) PDMS surfaces after 2-h adhesion is shown. ^∗∗∗^*p* < 0.0005, *t*-test, *n* > 3.

To verify if the defects/lack of stiffness-sensing and related phenotypes observed by the *oprF* mutant were not caused by any polar effect, pMH391-*oprF* was constructed to complement the *oprF* mutation. The complemented strain was studied following the same protocols used for the wild-type PAO1 and its *oprF* mutant. To specifically study the effects of *oprF*, the empty vector pMH391 (without *oprF*) was also electroporated into the wild-type *P. aeruginosa* PAO1 and its *oprF* mutant, and used as controls. As shown in **Figure 3**, insertion of this plasmid in PAO1 and its *oprF* mutant caused decrease in attachment for all samples compared to plasmid-free cells, presumably due to the metabolic burden caused by this high copy number plasmid. Nevertheless, complementation of *oprF* fully recovered the phenotypic changes in attachment, growth, and cell size observed in the *oprF* mutant. For example, the wild-type PAO1 carrying empty pMH391 (without *oprF*) exhibited around one log difference in the number of attached cells between soft and stiff PDMS surfaces. Such difference was not observed for the *oprF* mutant carrying empty pMH391 (without *oprF*) (*p* = 0.32, *t*-test, *n* = 3); and the complemented strain showed the normal one log difference in adhesion between soft and stiff surfaces as observed for the wild-type strain.

**FIGURE 4:**
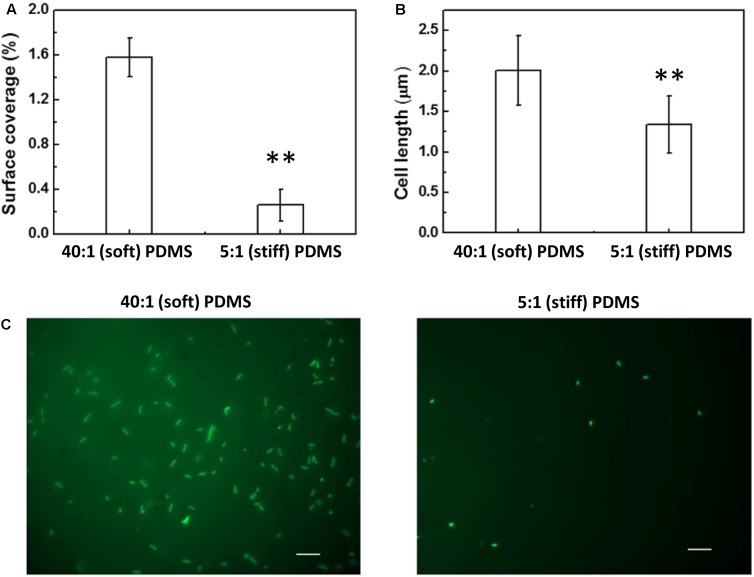
Effects of PDMS stiffness on the growth and cell size of the complemented *oprF* mutant. **(A)** Surface coverage of attached cells calculated using COMSTAT. **(B)** Average length of attached cells on soft (40:1 PDMS) and stiff (5:1 PDMS) surfaces. **(C)** Representative images of 5 h biofilm cells strained with acridine orange (Bar = 10 μm). ^∗∗^*p* < 0.005, *t*-test, *n* = 15 for surface coverage and *n* > 300 for cell length.

Complementation of the *oprF* gene also restored the difference between soft and stiff PDMS in biofilm cell growth and cell morphology as observed for the wild-type PAO1. As shown in **Figure 4**, there were more cells attached on soft PDMS surfaces than stiff PDMS surfaces, and cells being longer on soft PDMS, e.g., 2.0 ± 0.4 and 1.3 ± 0.4 μm on soft and stiff surfaces, respectively. These results showed that the defects in mechanosensing by the *oprF* mutant were recovered by complementation of the *oprF* gene and thus the defects observed in *oprF* mutant were not caused by any polar effect.

### The Role of Other *oprF*-Related Genes in the Mechanosensing of Material Stiffness

*oprF* encodes the major outer membrane surface porin protein OprF of *P. aeruginosa* to allow nonspecific solutes to pass cell membrane (Sugawara et al., 2006; Chevalier et al., 2017). OprF is also known to function for adhesion to animal cells (Azghani et al., 2002; Chevalier et al., 2017), and is involved in the secretion of several toxins, such as ExoT and ExoS (Wu et al., 2005), required for virulence (Fito-Boncompte et al., 2011). Moreover, three promoters of *oprF* are related to cell envelop stress, which are Pσ^70^, P*sigX*, and P*algU* (Bouffartigues et al., 2012). Absence of *oprF* was shown to abolish swarming and increase biofilm formation of *P. aeruginosa*, correlated with an increase in the level of intracellular c-di-GMP (Bouffartigues et al., 2011, 2015).

To further understand how *oprF* is involved in the response of *P. aeruginosa* to PDMS stiffness, several mutants of *oprF* related genes were compared for attachment on soft and stiff PDMS. The tested genes include *bifA*, *rpoS*, *rpoN*, *rhlA*, σ^70^, *sigX*, *fdxA*, *lecB*, and *exoT*. These genes were selected because they were reported to influence *oprF* expression or be influenced by the expression of *oprF* gene (Wu et al., 2005; Bouffartigues et al., 2011, 2012, 2015; Fito-Boncompte et al., 2011; Funken et al., 2012). We speculated that if *oprF* is involved in mechanosensing, mutations of related genes may also lose part of their ability to respond to PDMS stiffness. The *sigX* mutant showed a slight decrease in the difference in attachment between soft and stiff PDMS surfaces. For example, there were (1.9 ± 0.7) × 10^5^ cells/ cm^2^ and (4.7 ± 2.9) × 10^5^ cells/ cm^2^ on stiff and soft PDMS surfaces, respectively, after 2 h attachment. The difference is around half log, which is smaller than the one log difference exhibited by the wild-type PAO1. Since P*sigX* is the most critical one out of all the three promoters of *oprF* gene (P*sigX*, Pσ^70^, and P*algU*) (Bouffartigues et al., 2012), absence of *sigX* may reduce the expression level of *oprF* and thus affect mechanosensing. To verify if absence of *sigX* does affect *oprF* expression, we tested the transcription of *oprF* gene in *P. aeruginosa* PAO1 wild-type strain and its *sigX* mutant using qPCR. As expected, the results showed that *oprF* gene expression is 3.2 times higher in wild-type strain than its *sigX* mutant (*p* < 0.05, *t*-test, *n* = 3). Except for the *sigX* mutant, all the other mutants tested including *rpoN*, *lecB*, *fdxA*, *rhlA*, *bifA*, *rpoS*, σ^70^, and *exoT*, did not show any significant effect on adhesion (**Figure 1B**). The PAO1 mutant library does not have an *algU* mutant; however, by comparing a closely related wild-type *P. aeruginosa* PA14 (also exhibited marked difference in attachment between soft and stiff surfaces like PAO1) and its *algU* mutant, it was found that *algU* mutation did not abolish the response to PDMS stiffness (Supplementary Figure S2).

### Intracellular Cyclic-di-GMP Level Affected by Material Stiffness

**FIGURE 5:**
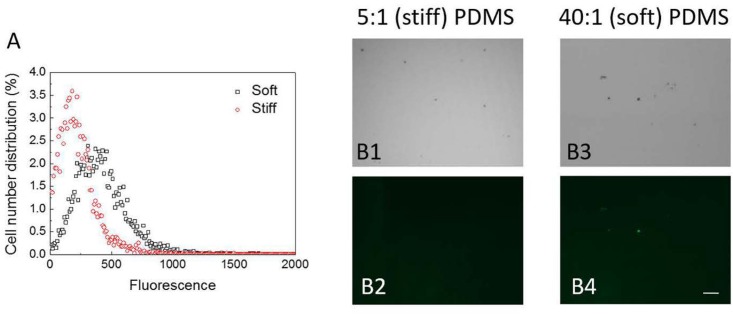
The intracellular c-di-GMP level in PAO1/pCdrA::*gfp*^s^ cells on soft (40:1) and stiff (5:1) PDMS surfaces after 2-h attachment. **(A)** Distribution of fluorescence signals measured by flow cytometry. **(B)** Representative images of PAO1/pCdrA::gfp(ASV)^s^ cells attached on soft (40:1) and stiff (5:1) PDMS surfaces after 2-h attachment (Bar = 10 μm). B1 and B3: bright field images. B2 and B4: green fluorescence images.

Cyclic dimeric guanosine monophosphate is a key factor regulating the transition from motile planktonic growth to biofilm formation (Hengge, 2009; Chua et al., 2015). Disruption of *oprF* has been shown to increase the intracellular level of c-di-GMP (Bouffartigues et al., 2015). Thus, we were curious if the level of c-di-GMP differs between cells on stiff and soft materials and if *oprF* mutation affects such difference. To answer these questions, we first compared the intracellular c-di-GMP level in the wild-type POA1 on soft and stiff PDMS surfaces, using a c-di-GMP reporter strain PAO1/pCdrA::*gfp*(ASV)^s^. With the *gfp* gene under the control of the promoter of *cdrA*, pCdrA::*gfp*(ASV)^s^ allows real-time monitoring of intracellular c-di-GMP level (Rybtke et al., 2012). After 2 h attachment the average intensity of the green fluorescence in PAO1/pCdrA::*gfp*^s^ cells on soft surfaces was found to be two times higher than that on stiff surfaces (*p* < 0.001, *t*-test; **Figure 5**). The distribution of green fluorescence in PAO1/pCdrA::*gfp*^s^ cells on soft surfaces was also wider than that on stiff surfaces (**Figure 5**). These results were corroborated by fluorescence microscopy, demonstrating that PAO1/pCdrA::*gfp*(ASV)^s^ attached to soft PDMS surfaces produced stronger green fluorescence than those on stiff surfaces (**Figure 5**). These findings indicated that intracellular c-di-GMP level is higher on soft PDMS than stiff PDMS during the 2 h attachment.

### *oprF* Mutation Increased the Level of Intracellular c-di-GMP

**FIGURE 6:**
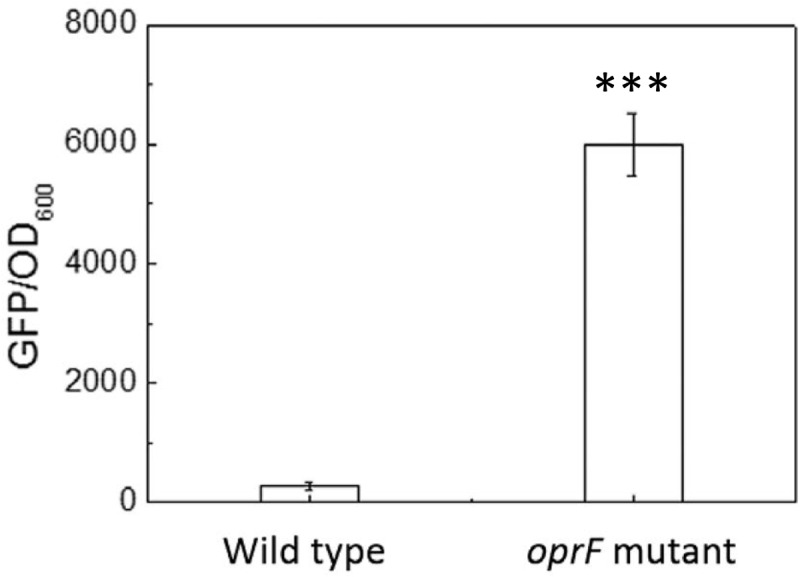
The intracellular c-di-GMP level in the wild-type *P. aeruginosa* and its *oprF* mutant. The plasmid pCdrA::*gfp*(ASV)^s^ was electroporated into both strains to monitor the intracellular c-di-GMP. ^∗∗∗^*p* < 0.0005, *t*-test, *n* = 3.

The finding that the intracellular c-di-GMP level is higher on soft PDMS suggests that c-di-GMP signal may be involved in mechanosensing by *P. aeruginosa* during attachment. To understand if *oprF* mutation affects the intracellular c-di-GMP, we compared the intracellular level of c-di-GMP between the wild-type PAO1 and its *oprF* mutant carrying the pCdrA::*gfp*(ASV)^s^ plasmid. The results in **Figure 6** showed the value of GFP/OD_600_ of wild type of *P. aeruginosa* and its *oprF* mutant strain measured by fluorescence spectrometer. The value of GFP/OD_600_ of *oprF* mutant (5952 ± 530) is significantly higher than the wild-type strain (265 ± 33) (*p* < 0.0001, *t*-test, *n* = 3), indicating that mutation of *oprF* coincided with an increase of intracellular c-di-GMP, which is consistent with the report by Bouffartigues et al. (2015). This result helps explain the increase in adhesion of *oprF* mutants on both soft and stiff PDMS (**Figure 3**), because high c-di-GMP level is known to promote adhesion (Chua et al., 2015).

We further compared the level of intracellular c-di-GMP in *oprF* mutant attached on soft and stiff PDMS. Unlike the wild-type POA1, *oprF* mutant showed no difference in intracellular c-di-GMP between soft and stiff PDMS (*p* > 0.4, *t*-test; Supplementary Figure S3). This is consistent with the finding that *oprF* abolished the difference in adhesion, growth, and cell length between soft and stiff PDMS.

Since *sigX* controls *oprF* expression, we also compared the level of intracellular c-di-GMP in *sigX* mutant attached on soft and stiff PDMS. The results showed that mutation of *sigX* substantially reduced the difference in the level of intracellular c-di-GMP between stiff and soft surfaces (Supplementary Figure S4) compared to the wild-type strain (**Figure 5**). This is expected since *sigX* controls *oprF* and *oprF* mutation abolished the response of *P. aeruginosa* to PDMS stiffness.

**FIGURE 7:**
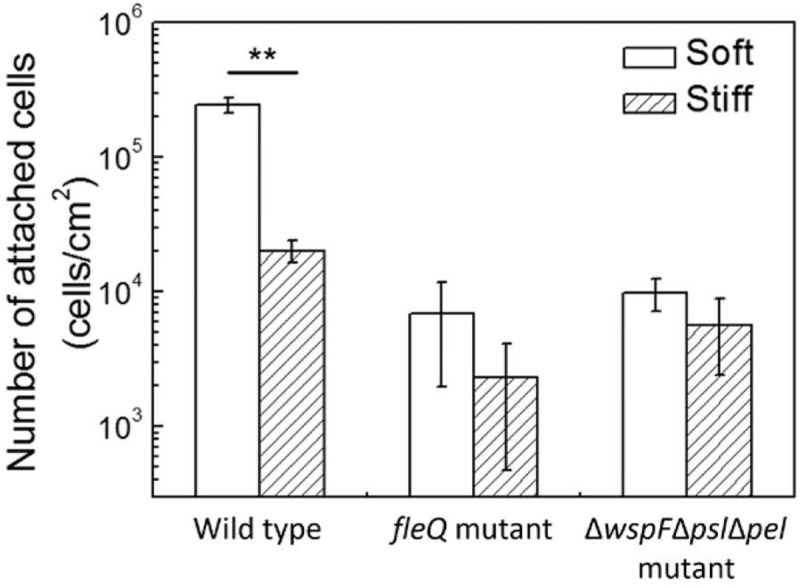
Effects of PDMS stiffness on attachment of the wild-type strain, its *fleQ* mutation strain, and Δ*wspF*Δ*psl*Δ*pel* mutation strain. The number of attached cells on soft (40:1) and stiff (5:1) PDMS surfaces after 2-h attachment is shown. Only the wild-type cells showed significant difference between stiff and soft surfaces. ^∗∗^*p* < 0.005, *t*-test, *n* > 6.

To further investigate if *oprF* mutation influences the mechanosensing of material stiffness through changes in the level of intracellular c-di-GMP, the *fleQ* mutant and Δ*wspF*Δ*psl*Δ*pel* triple mutant were compared for attachment on soft and stiff PDMS surfaces. These two mutants have been shown to have increased intracellular levels of c-di-GMP than the wild-type strain (Rybtke et al., 2012). FleQ is a regulator of flagella synthesis and can bind to c-di-GMP (Arora et al., 1997; Hickman and Harwood, 2008; Guttenplan and Kearns, 2013). By using the same reporter plasmid tested in this study, the values of GFP/OD_600_ of planktonic *P. aeruginosa fleQ* mutant and Δ*wspF*Δ*psl*Δ*pel* strain have been shown to be similar to the *oprF* mutant, much higher than that of the wild-type PAO1 (Rybtke et al., 2012). Consistently, we found the numbers of attached cells on soft and stiff PDMS were similar for both the *fleQ* mutant and Δ*wspF*Δ*psl*Δ*pel* (**Figure 7**), similar to the results of the *oprF* mutant and contrasting the wild-type PAO1. This finding indicates that high level of intracellular c-di-GMP may overpower the effects of material stiffness on cell attachment. It also suggests that the effects of *oprF* mutation on attachment may be through the changes in the intracellular level of c-di-GMP.

## Discussion

In this study, we found that *oprF* is involved in mechanosensing of material stiffness by *P. aeruginosa*. By changing the ratio of base:curing agent, the stiffness of PDMS was varied without significantly affecting other material properties. Jiang (2010) showed that the composition of C, O, and Si of PDMS including those included in this study using X-ray photoelectron spectroscopy (XPS) and found no difference among them. Moreover, Mata et al. (2005) reported that the types of PDMS surfaces used in this study have no significant change in hydrophobicity measured with surface contact angle. Previously we verified that these PDMS surfaces are not toxic to bacterial cells and do not affect the growth of *P. aeruginosa* (Song and Ren, 2014). Due to the different amounts of base and cross-linker, there is a possibility that the C=C on soft surfaces may be slightly more (maximum 5%) than those on stiff surfaces. However, this should be a minor effect since the maximum difference is only 5%, and it doesn’t change the hydrophobicity as mentioned above. In addition, the PDMS surfaces are essentially non-charged. Thus, the effects observed in this study are attributed to material stiffness.

Some previous studies have revealed how bacteria sense the physical contact with a surface (An and Friedman, 1998; Hickman et al., 2005; Petrova and Sauer, 2012; Belas, 2014). However, how stiffness of a surface material affects bacterial attachment at the genetic level is largely unknown. We recently reported that *motB* may be involved in the surface stiffness sensing by *E. coli* (Song et al., 2017). However, *motB* mutant did not show similar phenotype in *P. aeruginosa* (**Figure 1**). Since *E. coli* has multiple flagella per cell and *P. aeruginosa* is a single flagellum bacterium, the difference might be caused by how flagellar rotation and cell membrane–surface contact affect adhesion. Further studies using single cell level microscopy with high resolution to image flagella during attachment will be helpful.

A major finding of this study is that mutation of the *oprF* gene in *P. aeruginosa* PAO1 abolished its response to surface stiffness during attachment and the effects may involve changes in the intracellular c-di-GMP level. A higher level of intracellular c-di-GMP was found in the wild-type cells attached on soft PDMS surfaces than those on stiff surfaces, suggesting that material stiffness may affect bacterial biofilm formation through changes in the intracellular level of c-di-GMP, a key regulator that controls the switch from planktonic growth to biofilm formation. This also helps explain the effects of *oprF* mutation on the response of *P. aeruginosa* to PDMS stiffness. Since the *oprF* mutant exhibited around 300 times higher intracellular c-di-GMP than the wild-type strain based on the GFP signals (**Figure 6**), the change in c-di-GMP level due to PDMS stiffness is probably too small to affect biofilm formation. This is likely part of the mechanism why *oprF* mutation abolished the mechanosensing of surface stiffness. The results that *fleQ* mutants and Δ*wspF*Δ*psl*Δ*pel* mutants (also with high-level intracellular c-d-GMP) abolished the mechanosensing (**Figure 7**) support this hypothesis.

**FIGURE 8:**
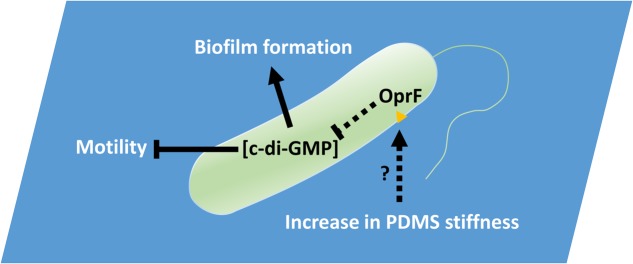
PDMS stiffness affects *P. aeruginosa* biofilm formation. Material stiffness may be sensed by OprF, either directly or indirectly, and consequently affect the intracellular level of c-di-GMP. Thus, on a surface with desired mechanical cues, the cells will have an increased level of c-di-GMP, which will reduce motility and induce biofilm formation as observed on soft PDMS. In contrast, if the material stiffness is undesired, the c-di-GMP level may remain low and cells will retain rather high-level motility, which delays or inhibits biofilm formation.

To attach on a surface, bacteria need to overcome any repulsion from the surface and replace the solid–liquid interface with cell–solid interface. Cell membrane deformation is also required for attachment to take place (Harapanahalli et al., 2015a,b). This will likely cause a stress to the cell membrane. Since OprF is an important membrane component and is involved in cell shape control (Rawling et al., 1998), it is possible that the stress can be sensed by OprF, either directly or indirectly, and consequently affect the intracellular level of c-di-GMP. We speculate that bacteria membrane deformation, as part of requirement for attachment, creates more membrane stress to cells on a hard PDMS surface than those on a soft one, which is sensed by OprF. When the stress is high, it lowers the production of c-di-GMP and thus delays biofilm formation. On a soft surface, the deformation is easier to happen, leading to the opposite phenomenon, e.g., higher c-di-GMP level and more biofilm formation. Consistently, when *oprF* is mutated, the level of c-di-GMP increases and the cells make more biofilms even on hard surfaces as observed in this study. Thus, on a surface with desired mechanical cues, the cells will have an increased level of c-di-GMP, which will reduce motility and induce biofilm formation as observed on soft PDMS. In contrast, if the material stiffness is undesired, the c-di-GMP level may remain low and cells will retain rather high-level motility, which delays or inhibits biofilm formation (**Figure 8**). Recently, Rodesney et al. (2017) reported that c-di-GMP is also responsible for the mechanosensing of shear force of flow. This provides additional evidence that bacteria can sense mechanical cues and adjust physiology by changing the intracellular level of c-di-GMP. The response may also involve FleQ since it binds to c-di-GMP and regulates flagellar synthesis and pel/psl production, all are important to biofilm formation. How exactly the signal of material stiffness is transduced and causes the change in c-di-GMP level is unknown. We tested a number of mutants in this study (described in the section “Results”) and more in a parallel study in *P. aeruginosa* PA14 (the result of *ΔdgcB* mutant, as an example, is shown in Supplementary Figure S2; others are not shown), but only *oprF* of PAO1 fully abolished the response to PDMS stiffness. The lack of distinct phenotypes of other mutants is not surprising. Compared to general contact with a surface, which is planktonic vs. attached cells, stiffness varies at different levels and subtler changes (and more genes and pathways) may be involved in the surface stiffness sensing/responding. More quantitative studies are required as follow-on work to fully elucidate the mechanism. Other factors such as surface chemistry and charge should also be taken into consideration when comparing results from different systems.

## Conclusion

In this study, we demonstrated that mutation of *oprF* abolished the response by *P. aeruginosa* to material stiffness during attachment and early biofilm formation on PDMS surfaces, which was rescued by complementing the *oprF* gene. Since the level of intracellular c-di-GMP in the attached wild-type cells was higher on soft PDMS than stiff PDMS and the mutation of *oprF* led a significant increase in the intracellular c-di-GMP, it is possible that OprF plays a role in response to material stiffness by affecting the level of c-di-GMP. This is consistent with the results of another two mutants, *fleQ* and Δ*wspF*Δ*psl*Δ*pel* triple mutant, which have similar high levels of c-di-GMP as the *oprF* mutant. These two mutants also lost the capability to differentiate PDMS stiffness. Overall, this study provides new evidence that bacteria can sense and respond to the stiffness of substrate material as a mechanical cue during attachment and reveals that *P. aeruginosa oprF* and c-di-GMP can influence such response.

## Author Contributions

FS and DR conceived and designed the experiments. FS and HW performed the experiments. FS, HW, KS, and DR analyzed the data. All authors participated in paper writing and revision.

## Conflict of Interest Statement

The authors declare that the research was conducted in the absence of any commercial or financial relationships that could be construed as a potential conflict of interest.
