# Supplementary material for: Cyclic-di-GMP and oprF Are Involved in the Response of Pseudomonas aeruginosa to Substrate Material Stiffness during Attachment on Polydimethylsiloxane (PDMS)
Source: Front Microbiol. 2018 Feb 1;9:110. doi: 10.3389/fmicb.2018.00110 (PMC5799285; doi:10.3389/fmicb.2018.00110)
Supplement: Supplementary file 1 [file Presentation_1.pdf]

Supporting Information

for

Cyclic-di-GMP and *oprF* are involved in the response of *Pseudomonas aeruginosa* to the stiffness of polydimethylsiloxane (PDMS) during attachment on polydimethylsiloxane (PDMS)

Fangchao Song[1,2,†], Hao Wang[1,2,†], Karin Sauer,[3] Dacheng Ren[1,2,4,5,*]

[1]Department of Biomedical and Chemical Engineering, Syracuse, New York, USA.

[2]Syracuse Biomaterials Institute, Syracuse, New York, USA.

[3]Department of Biological Science, Binghamton University, Binghamton, New York, USA.

[4]Department of Civil and Environmental Engineering, Syracuse, New York, USA.

[5]Department of Biology, Syracuse University, Syracuse, New York, USA.

[†] These authors contributed equally.

***Corresponding author:**

Dacheng Ren: Phone +1-315-443-4409. Fax +1-315-443-9175. Email : dren@syr.edu

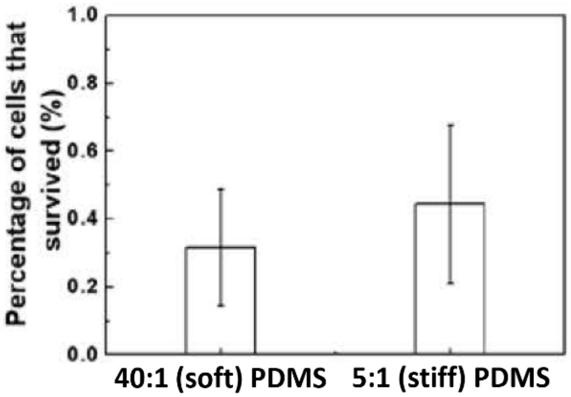

Figure S1. Survival of *P. aeruginosa* PAO1 *oprF* mutant after treatment with 20 µg/mL tobramycin for 3.5 h. The difference is insignificant ($p > 0.05$, t test, n=6).

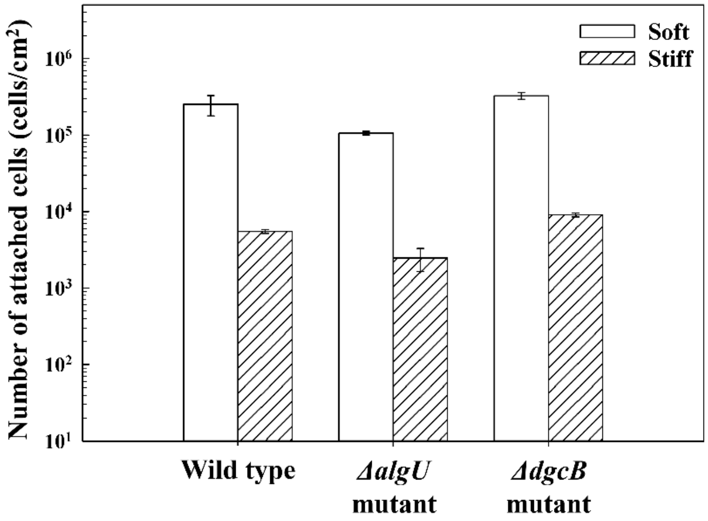

Figure S2. Effects of PDMS stiffness on 2 h attachment of *P. aeruginosa* PA14 (wild type) and its *ΔalgU* and *ΔdgcB* mutants on soft and stiff PDMS.

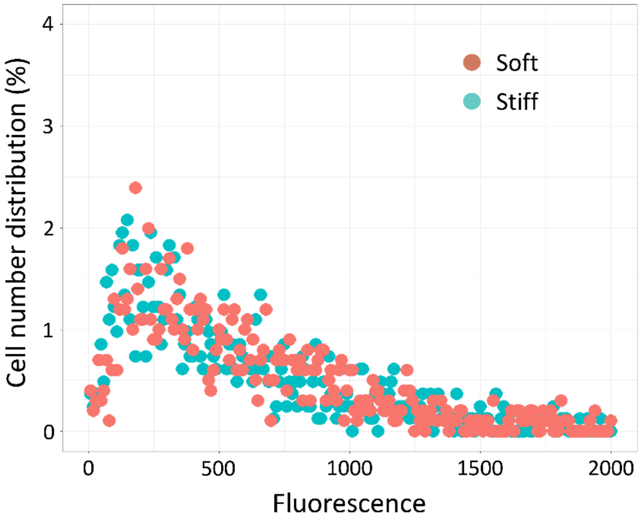

Figure S3. Florescence (reporter of intracellular c-di-GMP level) of *P. aeruginosa* PAO1 *oprF*/pCdrA::*gfp*^{s} cells on soft (40:1) and stiff (5:1) PDMS surfaces after 2-h attachment. The distribution of fluorescence signals was measured by flow cytometry.

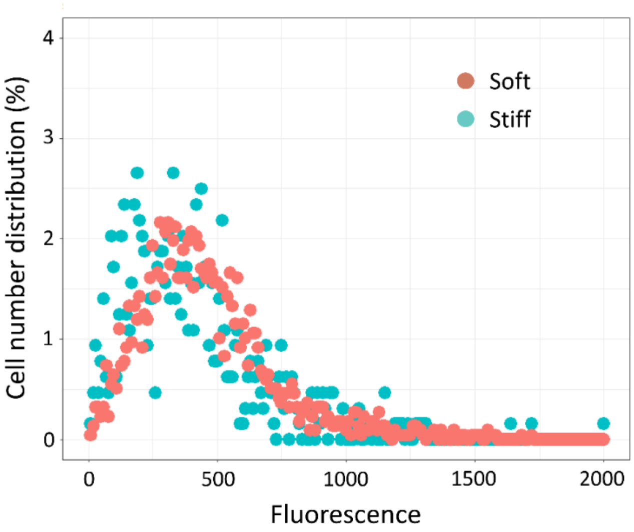

Figure S4. Florescence (reporter of intracellular c-di-GMP level) of *P. aeruginosa* PAO1 *sigX*/pCdrA::*gfp*^{s} cells on soft (40:1) and stiff (5:1) PDMS surfaces after 2-h attachment. The distribution of fluorescence signals was measured by flow cytometry.
